# Combination of a DC Motor Controller and Telemetry System to Optimize Energy Consumption

**DOI:** 10.3390/s23156923

**Published:** 2023-08-03

**Authors:** Paweł Żur

**Affiliations:** Department of Engineering Processes Automation and Integrated Manufacturing Systems, Faculty of Mechanical Engineering, Silesian University of Technology, 44-100 Gliwice, Poland; pawel.zur@polsl.pl

**Keywords:** motor, controller, Android, energy saving, telemetry

## Abstract

The paper introduces the development stages of a MOSFET-based controller for a DC brush motor. The main objective was to design a controller that could be integrated with the existing telemetry system, offering full configurability through an Android application. This controller aims to provide real-time analysis of data collected from the measurement system, including motor revolutions and current draw. Based on the analyzed data and the conditions set in the Android application, the controller adjusts the motor’s operational characteristics accordingly. The paper provides a comprehensive description of the controller system’s functioning. The proposed control system is particularly relevant in applications where minimizing energy consumption for driving a DC motor is of utmost importance.

## 1. Introduction

In today’s fast-changing world, saving energy and improving how things work is vitally important. One area where this could have the greatest impact is in the electric motors used in various industries like transportation, manufacturing, and renewable energy [1,2,3]. Within these industries, electric motors play a vital role, driving progress and revolutionizing traditional power systems. However, harnessing the full potential of electric motors requires sophisticated control mechanisms capable of fine-tuning their operations [4,5].

This paper presents a motor controller capable of optimizing motor performance in real time, with a particular focus on integrating electric motor controllers with telemetric systems to enhance energy consumption [4,6].

The telemetric system connected to an Android application plays a major role in that integration. This powerful combination provides the means of monitoring critical motor parameters, such as the current drawn from the motor and the revolutions per minute (RPM) of the motor, with the utmost precision and accuracy. The telemetric system acts as a digital conduit, transmitting invaluable information to the motor controller in real time, enabling seamless adjustments and optimizations [7,8].

Despite the promising advancements in electric motor controllers and telemetry systems for optimizing energy consumption, there remains a significant research gap in exploring the full potential of their integration within the context of electric car competitions. While the existing literature acknowledges the benefits of using PWM signals for controlling motor behavior and enhancing energy efficiency, there is limited research on customizing these systems for specific predefined conditions. One of the primary objectives of an electric motor controller is to achieve the highest level of energy efficiency. By dynamically adjusting the control signals sent to the motor, the controller optimizes the motor’s power consumption, ensuring that energy is utilized effectively without compromising performance. The key to accomplishing this lies in the implementation of Pulse Width Modulation (PWM) signals [9,10,11].

The controller configures the PWM signal according to the real-time data received from the telemetric system, carefully balancing power output and motor speed. By modulating the duty cycle of the PWM waveform, the controller can achieve precise control over the motor’s behavior. This ability to fine-tune the power delivered to the motor results in reduced energy waste and increased overall efficiency [10,11,12].

The FRACMO 624-65-112 motor is used in the electric car used by the Silesian Greenpower team. This team participates in international competitions for electric cars. The goal of the competition is to drive as great a distance as possible in one hour, using only two 12 V batteries with a capacity of 36 Ah each. Therefore, a motor controller that can adapt to the actual track conditions is a key element [7,8,13]. 

In conclusion, the electric motor controller, when paired with a telemetric system and harnessed through an Android application, represents a potent synergistic device for achieving optimal energy consumption and maximizing the performance of electric motors. By addressing the specific requirements of electric car competitions, this research not only advances the understanding of energy-efficient systems but also offers practical implications for real-world applications. The findings of this study are expected to have broader implications for electric vehicle technology and sustainable transportation, paving the way for further advancements in the field. Ultimately, this article serves as a comprehensive guide, unravelling the complexities of this integration, and highlighting the pivotal role played by the controller in driving the future of energy-efficient systems.

## 2. Materials and Methods

### 2.1. Motor Controller and Integration with a Telemetric System

In order to control a DC motor, a system consisting of the following components was designed:

- Step-down voltage converter set to 15 V output voltage;
- MCP1407 MOSFET control circuit;
- EL817C optocoupler;
- MOSFET circuit.

**Figure 1:**
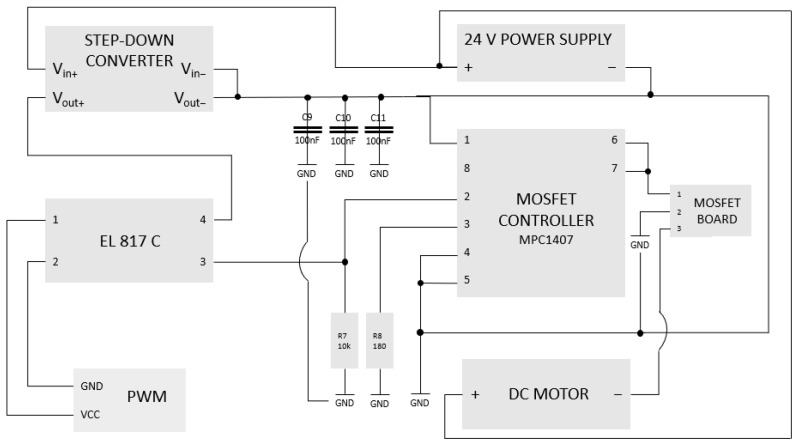
Wiring diagram of the motor controller.

The wiring diagram for the above components is presented in Figure 1 below.

The EL817C optocoupler was added to the circuit due to the need to separate the ground of the measurement and control circuit. The DC motor generates significant interference, which caused blocking of the USB OTG port on the phone connected to the measuring circuit. Stalica et al. [7] described the telemetry system that is currently being used for measurements in the car. The designed measuring system allows us to preview the motor operation parameters during the race in real time. It enables the correct selection of the transmission ratio to the current track conditions. An important element of the measurement system is the current measurement sensor. For this purpose, the HTFS-200P sensor was used. The basic advantage of measuring the current with this sensor is that its operation is based on the use of the Hall effect. The installation of the sensor does not interfere with the high-voltage circuit but only limits it to inserting the cable of which we want to measure current through the hole in the sensor.

**Figure 2:**
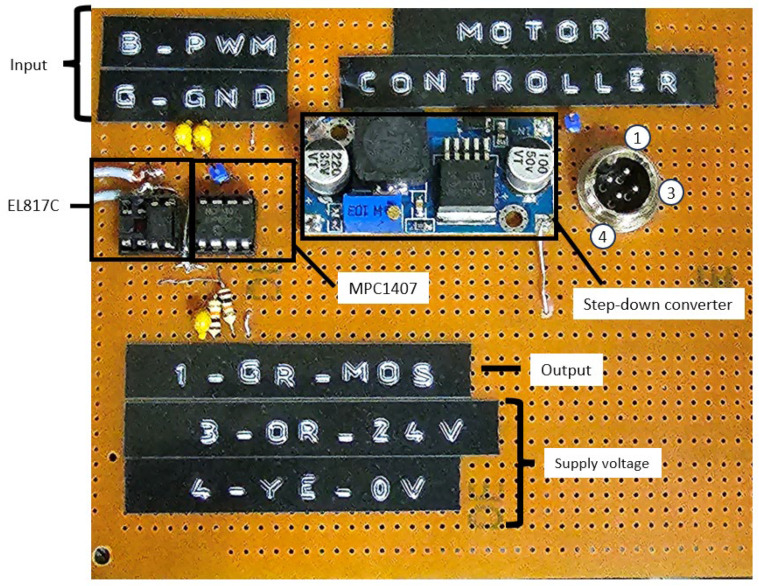
The circuit of the motor controller.

An existing telemetry solution was used to generate the PWM signal controlling the MCP1407 chip. The finished circuit is shown in Figure 2.

The figure shows the individual elements of the circuit described earlier in the wiring diagram. The numbers 1, 3, and 4 indicate the connector. Pin 1-GR-MOS corresponds to the signal output to the MOSFET. Pin 3-OR-24V is responsible for the added pole of the supply voltage. Pin 4-YE-0V corresponds to the negative pole of the power supply. The B-PWM designation refers to the blue wire connecting the measurement circuit (PWM signal), while G-GND refers to the ground wire from the telemetry circuit.

### 2.2. C++ Code Implementation for Dynamic PWM Control

**Figure 3:**
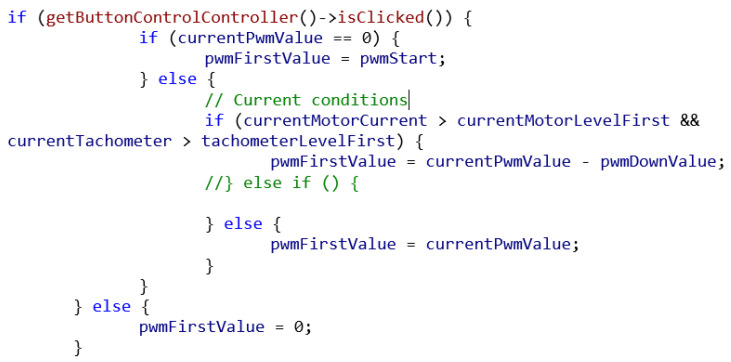
Sample of the code implementation.

The C++ code utilizes the data obtained from the telemetric system, including the RPM and current measurements, to calculate and update the PWM value in real time. On the basis of the acquired RPM and current values, the code calculates the desired PWM value. This calculation can be customized based on the specific control algorithm and optimization strategy used. The calculated PWM value is then sent to the motor controller to update the PWM signal. This ensures that the motor receives the appropriate power input based on the current operating conditions. The code snippet presented in Figure 3 below provides a basic foundation for dynamic PWM control based on the RPM and current measurements. However, it is important to note that the actual implementation may vary depending on the specific requirements of the motor and the control strategy used.

Conditions were implanted in the code to prevent the user from drawing more than 18A of current during any particular trip. This value is conventional and can be dynamically changed, requiring further analysis and customization for a specific user’s needs. The software also checks the actual speed of the motor, by which it tries to keep it within the optimal RPM range according to the motor manufacturer’s data sheet [14].

### 2.3. Measuring Station Description

**Figure 4:**
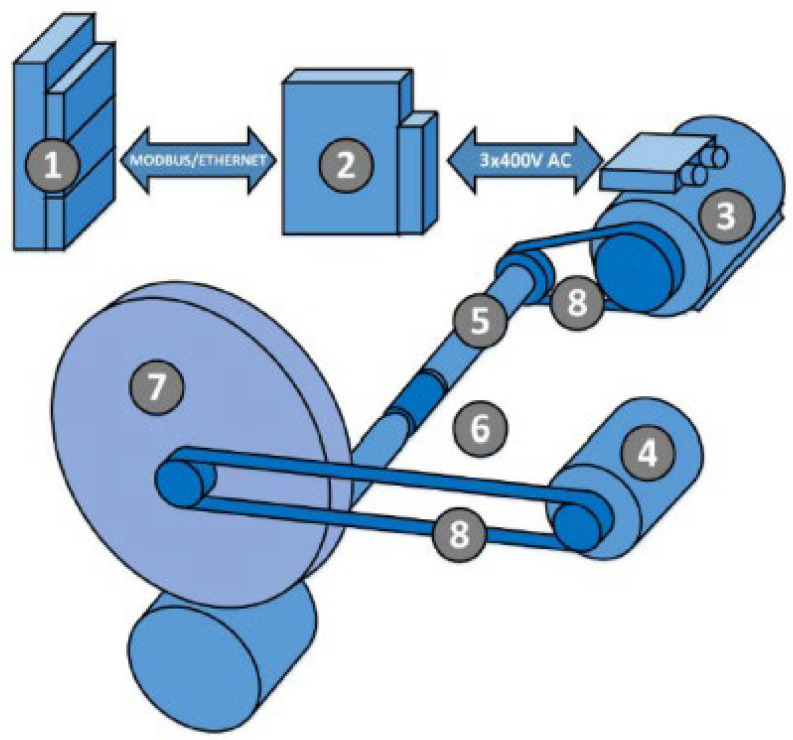
Simplified drawing of the dyno test stand.

To investigate the performance and efficiency of the electric motor controller, a dedicated measuring station was employed. A schematic view of the dyno test stand is presented in Figure 4 below.

The elements presented in the schematic view are described below:

1. PC and router;
2. MDX61B frequency converter with DFE11B expansion card;
3. DRE80M4 three-phase asynchronous motor with incremental encoder;
4. DC electric motor (used in a vehicle);
5. Shaft with mass wheel;
6. NCTE2200-75 Nm torque sensor with CompactRIO platform NI9149;
7. Vehicle wheel;
8. Toothed belts.

This station comprised a DC motor coupled with an SEW drive acting as a load, allowing for precise measurements of the key parameters. The SEW drive played a crucial role in capturing the torque exerted by the motor on a roller, which was connected via a toothed belt and wheel mechanism.

### 2.4. SEW Drive and Torque Measurement

**Figure 5:**
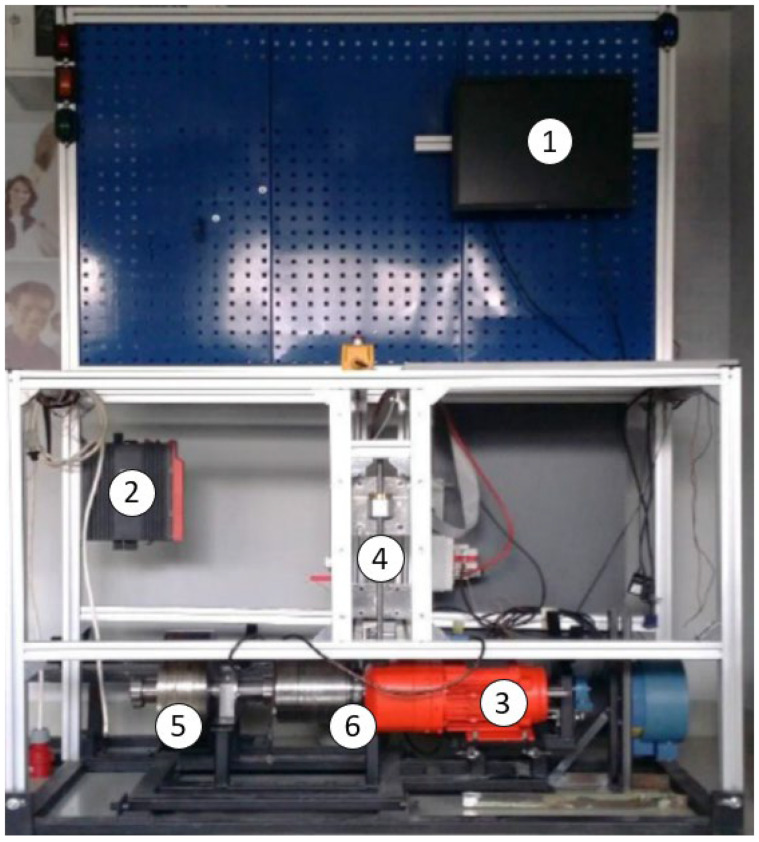
Picture of the dyno test stand.

The SEW drive utilized in the measuring station offered a reliable and accurate method of quantifying the torque generated by the DC motor. By measuring the torque, it was possible to assess the motor’s output power and its ability to drive various loads. A view of the SEW drive dyno test stand is presented in Figure 5 below. The numbers in the picture correspond to the elements of the test stand listed in the previous section. The vehicle wheel and toothed belts are not presented in the picture.

The timing belt and wheel arrangement served as the mechanical connection between the motor and the SEW drive. As the motor rotated, the toothed belt transmitted the rotational force to the wheel, which, in turn, applied torque to the roller. This configuration allowed the SEW drive to measure the torque accurately and in real time.

### 2.5. Application in LabVIEW for Load Diagram Configuration

**Figure 6:**
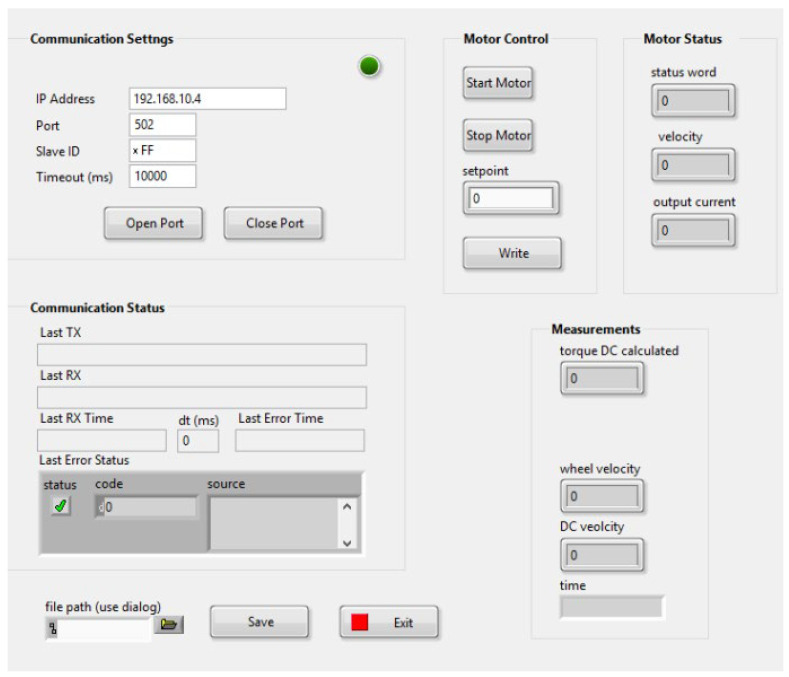
LABView application view.

To simulate different load scenarios and evaluate the performance of the electric motor controller under various conditions, a LabVIEW application was employed. LabVIEW, a powerful graphical programming environment, allows for the creation of custom control interfaces and data acquisition systems. The LabVIEW application utilized in this study facilitated the configuration of the load diagram, allowing precise control over the load parameters. In the application, it is possible to define and adjust the load characteristics, such as torque levels and variations, to replicate real-world operating conditions and assess the controller’s ability to optimize energy consumption accordingly. A view of the application window is presented in Figure 6 below.

The application allows us to generate an additional load on a DC motor using the aforementioned SEW motor. By assigning a ‘Setpoint’ value, it is possible to change the torque value with which the motor loads the test stand. Generating a variable load allows us to verify the behavior of the DC motor control system in the event of a change in the current drawn from the batteries.

### 2.6. Data Acquisition and Analysis

**Figure 7:**
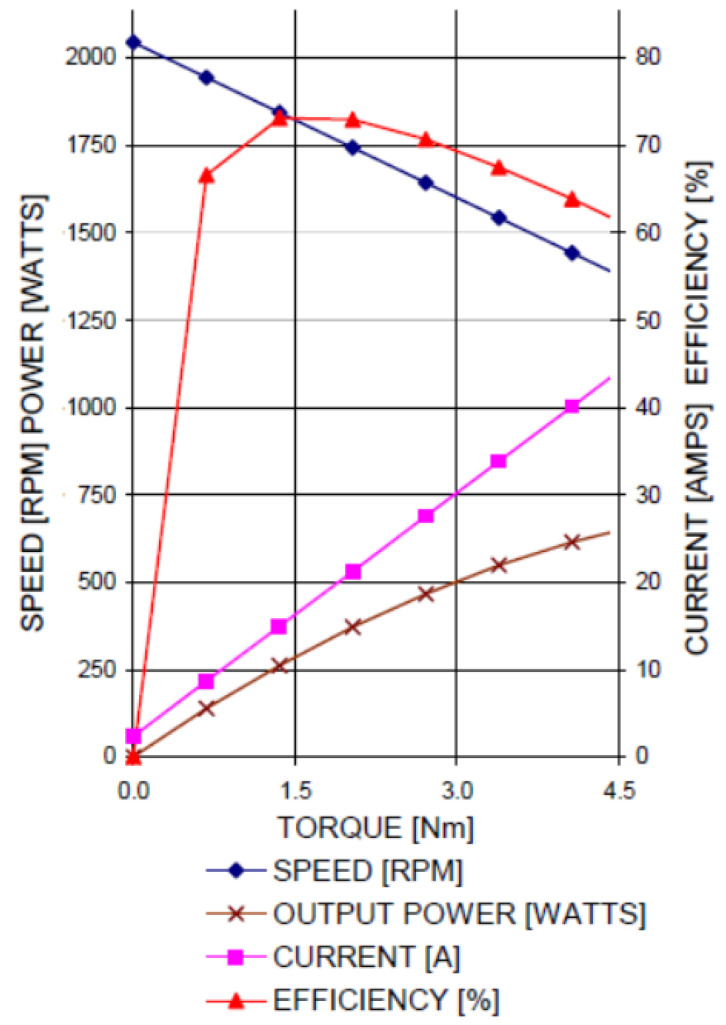
Performance graph from the Fracmo 24-65-112 motor datasheet [14].

The data collected were subsequently processed and analyzed using statistical and graphical tools. These findings served as a basis for evaluating the controller’s efficiency in optimizing energy consumption and its ability to adapt to varying load demands. It should be noted that this specific DC motor has the highest efficiency at approximately 1800 RPM, as presented in Figure 7. 

During the experiments, different load profiles were applied using the LabVIEW application, mimicking various operational scenarios. The motor’s performance was continuously monitored, and the corresponding data were recorded for subsequent analysis. Through this set-up, the torque exerted by the motor and the load characteristics were precisely controlled and measured, while the telemetric system and the motor controller enabled real-time adjustments to optimize energy consumption.

## 3. Results

In this chapter, we discuss the findings and outcomes of our research on the adaptive electric motor controller. As the demand for efficient and sustainable transportation continues to increase, electric motor controllers play a crucial role in optimizing the performance and energy consumption of electric vehicles and industrial machinery. The results are presented and discussed below.

### 3.1. Current Measurements

One of the key parameters monitored during the experimental trials was the current drawn from the motor over time. Current values were recorded using the telemetric system connected to the motor controller and were recorded at regular intervals throughout the testing period. 

**Figure 8:**
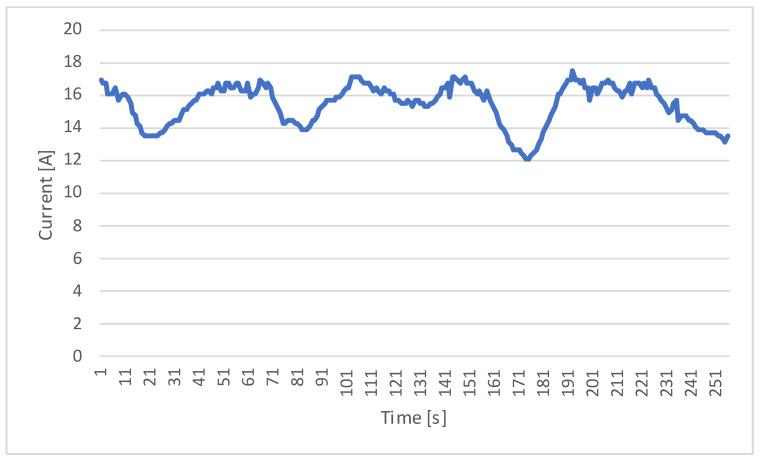
Current measurements over time.

Figure 8 illustrates the trend in the current values over time, showcasing the dynamic behavior of the motor’s power consumption. The current values range from 12 to 18 amps, demonstrating the varying load demands experienced by the motor during the experimental trials.

The measurements taken show the stage where the motor has already accelerated to its nominal speed. The acceleration stage itself should be a stage of separate analysis and testing.

According to the program, the current value did not exceed 18A.

### 3.2. RPM Measurements

Another critical parameter monitored during the experiments was the revolutions per minute (RPM) of the motor. RPM values were also recorded using the telemetric system connected to the motor controller and logged at regular intervals throughout the testing period. 

**Figure 9:**
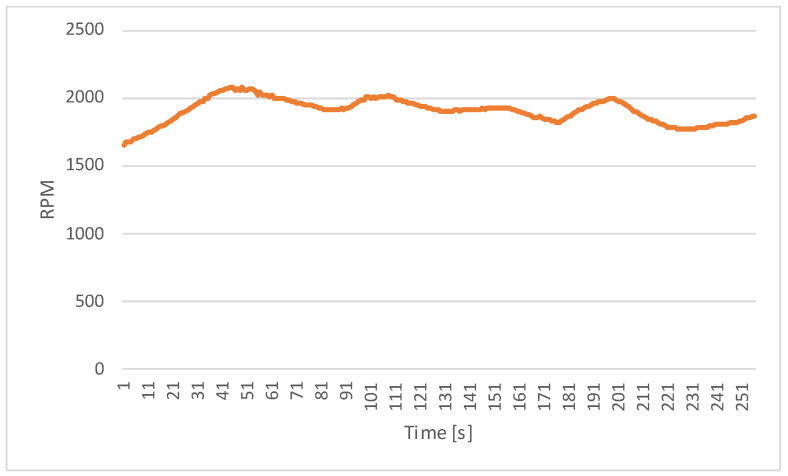
RPM measurements over time.

Figure 9 displays the RPM values over time, highlighting the motor speed variations throughout the experimental trials. The RPM values range from 1650 to 2100, reflecting the motor’s ability to adjust its rotational speed to effectively meet the load demands.

In the above graph, it can be seen that the controller attempted to keep the motor within the optimal speed range.

### 3.3. Current and RPM Correlation

**Figure 10:**
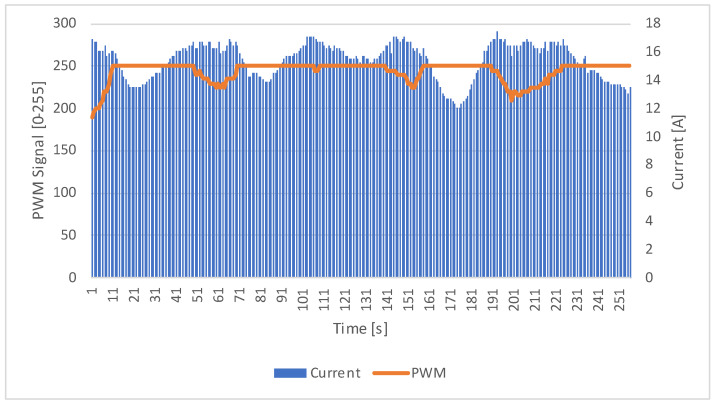
Current and PWM correlation.

The purpose of the present study was to enable the PWM signal controlling the motor to be dynamically varied in fill, based on measured values in real time. Figure 10 illustrates the relationship between the current and the PWM signal over time, allowing for a comprehensive analysis of the motor behavior under different load conditions.

This correlation signifies the motor controller’s ability to optimize power delivery, ensuring that the motor responds to load variations by adjusting its rotational speed accordingly.

Overall, the results presented here provide a comprehensive understanding of the motor’s performance in terms of the current draw and RPM. The analysis of these parameters is crucial in evaluating the effectiveness of the electric motor controller and its ability to achieve optimal energy consumption and performance. 

The final step compared the battery current during the test with the same set variable load. For the test, the controller designed in the present work and a purchased controller available on the market were considered. The test parameters were as follows:

1. Test time: 120 s;
2. Load applied: 0.4 Nm;
3. Intervals of inflicted load: 30:10 (30 s of load, 10 s of no load).

The load scheme is based on real, albeit simplified, movement on a section of track where, during a race, the car makes three turns with a 3:1 ratio of load (current is drawn from the batteries) to no load (current is not drawn, the car rolls).

Table 1 shows the current consumed by the batteries during the test run.

The power consumption in the test conducted showed that the designed controller is more energy efficient. Almost 10% less energy was consumed, which, for the electric car in which the controller will be used, is a clear outcome that could affect the final result.

## 4. Discussion

In this chapter, the results of the research on an adaptive motor controller are discussed. This specific controller is capable of adjusting itself as the motor’s conditions change, allowing the electric motor to work in the optimal RPM range and more reliably in general. By looking at the experiment results, studying the advantages and limitations, and exploring how it can be used, it can be understood how adaptive motor controllers can really change the way we control electric motors.

### 4.1. Interpreting the Results in the Context of Previous Studies

The results obtained from the current and RPM measurements provide valuable insight into the performance of the electric motor controller and its impact on energy consumption. These findings can be interpreted within the context of previous studies and work hypotheses, shedding light on the effectiveness of the controller in achieving optimal efficiency.

Previous studies have highlighted the importance of fine-tuning the control signals sent to the motor to minimize energy waste and maximize performance. The current measurements demonstrate that the motor controller successfully adjusts the power delivery based on the load demands. As the current draw increases, the RPM of the motor also increases, indicating that the controller effectively modulates the PWM signal to optimize energy consumption while meeting the required load.

Furthermore, the observed correlation between the current and RPM aligns with the working hypotheses, reinforcing the notion that the motor controller adjusts the motor’s speed in response to varying load conditions. This dynamic adjustment ensures that the motor operates in an optimal RPM range, allowing for efficient energy utilization while maintaining performance.

### 4.2. Implications and Broader Context

The results of this study show that electric motor controllers have great potential to improve energy efficiency solutions in many industries. By integrating telemetric systems and smart control algorithms, the controller can optimize energy consumption and improve electric motors’ work overall. These improvements could be beneficial for various fields and industries.

Optimizing energy consumption in electric motors is crucial to reduce greenhouse gas emissions and promote sustainability. The results demonstrate that the motor controller, through real-time adjustments of the PWM signal, can effectively balance power output and motor speed to achieve optimal energy efficiency. This has significant implications for applications ranging from electric vehicles to industrial machinery, where energy conservation plays a vital role.

Furthermore, the successful integration of telemetric systems and the LabVIEW application highlights the importance of real-time data acquisition and analysis. This enables researchers and motorists to monitor and fine-tune motor performance, facilitating continuous improvements in energy efficiency and operational effectiveness.

### 4.3. Future Research Directions

Based on the findings of this study, several avenues for future research can be identified. First, further investigations could focus on optimizing control algorithms within the motor controller. To improve the energy optimization algorithm, optimization techniques such as ALO or EHO could be compared. Fine-tuning these algorithms could lead to even more precise adjustments of the PWM signal, resulting in enhanced energy efficiency and performance.

Additionally, exploring the integration of machine learning and artificial intelligence techniques into the motor controller could provide additional opportunities for efficiency improvement. Using data-driven approaches, the controller’s performance could be adapted and optimized based on real-time feedback and predictive models, leading to more sophisticated and intelligent motor control systems.

Furthermore, expanding the scope of the study to evaluate the controller performance under different load profiles and operating conditions could provide a comprehensive understanding of its capabilities. Examining the controller’s response to dynamic and transient loads could further enhance its performance and energy efficiency.

Lastly, investigating the scalability and applicability of the motor controller in larger-scale systems and complex network environments would be a valuable area of research. Understanding how the controller interacts with multiple motors and its integration into smart grid systems could unlock further possibilities for energy optimization and system-wide efficiency improvements.

## 5. Conclusions

In conclusion, the findings of this study demonstrate the effectiveness of the electric motor controller in optimizing energy consumption and enhancing motor performance. By integrating telemetric systems, advanced control algorithms, and the LabVIEW application, the controller successfully adjusts the PWM signal based on real-time data, achieving optimal energy efficiency and adapting to varying load demands. Furthermore, the successful implementation of the electric motor controller and its ability to optimize energy consumption contribute to social welfare by promoting sustainability and reducing environmental impact. As electric vehicles become more prevalent, the adoption of energy-efficient motor control technologies could significantly reduce greenhouse gas emissions and decrease the overall carbon footprint. By enhancing the performance and energy efficiency of electric motors, this research can foster a broader societal transition towards cleaner and greener transportation solutions. Additionally, the findings of this study hold relevance beyond the electric vehicle industry, as improved motor control technologies can be applied in various sectors, such as industrial automation and renewable energy systems, further benefiting society as a whole. The positive impact on the environment and the potential for wider application underscore the importance of advancing motor control technologies to align with the global goals of sustainable development and responsible resource management. The conclusions can be summarized in the following points:

- The proposed circuit board on the MCP1407 chip fulfilled its role and was successfully implemented.
- The EL817C optocoupler is necessary to avoid the interference caused by the DC brush motor.
- The assumed method of cooperation of the telemetry system together with the motor controller was successfully validated. The controller is able to change the motor’s operating characteristics in real time, which has a beneficial effect on saving the current drawn from the batteries. The test carried out showed that almost 10% lower power consumption was achieved during the interval loading of the motor on the designed controller.
- As part of the next research, a simulation should be carried out considering the entire 1 h race.

## Figures and Tables

**Table 1:** Current during the test run.

Type of Controller	Current Drawn [Ah]	Avg Current [A]
Customizable motor controller	0.5114	15.34
Other stock motor controller	0.5521	16.56
